# Corrosion Behavior of Silver-Plated Circuit Boards in a Simulated Marine Environment with Industrial Pollution

**DOI:** 10.3390/ma10070762

**Published:** 2017-07-06

**Authors:** Kui Xiao, Pan Yi, Lidan Yan, Ziheng Bai, Chaofang Dong, Pengfei Dong, Xiong Gao

**Affiliations:** Corrosion and Protection Center, University of Science and Technology Beijing, Beijing 100083, China; B20160547@xs.ustb.edu.cn (P.Y.); S20140516@xs.ustb.edu.cn (L.Y.); G20169110@xs.ustb.edu.cn (Z.B.); cfdong@ustb.edu.cn (C.D.); G20159099@xs.ustb.edu.cn (P.D.); S20151321@xs.ustb.edu.cn (X.G.)

**Keywords:** silver-plated circuit boards, simulated polluted environment, electrochemical impedance spectroscopy, potentiodynamic polarization curves

## Abstract

The electrochemical corrosion behavior of a silver-plated circuit board (PCB-ImAg) in a polluted marine atmosphere environment (Qingdao in China) is studied through a simulated experiment. The morphologies of PCB-ImAg show some micropores on the surface that act as the corrosion-active points in the tests. Cl^−^ mainly induces microporous corrosion, whereas SO_2_ causes general corrosion. Notably, the silver color changes significantly under SO_2_ influence. EIS results show that the initial charge transfer resistance in the test containing SO_2_ and Cl^−^ is 9.847 × 10^3^, while it is 3.701 × 10^4^ in the test containing Cl^−^ only, which demonstrates that corrosion accelerates in a mixed atmosphere. Polarization curves further show that corrosion potential is lower in mixed solutions (between −0.397 V SCE and −0.214 V SCE) than it in the solution containing Cl^−^ only (−0.168 V SCE), indicating that corrosion tendency increases with increased HSO_3_^−^ concentration.

## 1. Introduction

Currently, electronic components are widely used. However, these components are susceptible to corrosion, especially in environments that contain moisture, dust, and atmosphere pollutant [1,2]. Chlorine (Cl^−^) and sulfur dioxide (SO_2_) are significant contaminants in the environment that can lead to high corrosion rates of silver and copper [3,4].

Copper and silver are sensitive to Cl^−^ [5,6,7]. In recent years, the effects of Cl^−^ on the corrosion mechanism of copper have been studied [8,9,10,11,12]. In a solution containing Cl^−^, CuCl forms rapidly on the surface of copper; this film later transforms into CuCl_2_^−^, and protection of the interior copper atoms is lost with continuous penetration of chloride ions [13,14]. The anodic dissolution is determined by the rate of dissolution of the CuCl_2_^−^ into bulk [14,15]. AgCl usually forms on the surface of silver in physiological NaCl solution [16]. Moreover, a high concentration of Cl^−^ in the electrolyte film accelerates the dissolution of AgCl [17,18,19]. 

SO_2_ is one of the main corrosion factors for copper and silver. SO_2_ can be adsorbed in the thin electrolyte film on the surface of copper and silver when exposed to a humid environment. Studies have found that these presently formed corrosion products, such as Ag_2_O, AgOH, and Ag_2_SO_3_, act as physical barriers that protect the electrode surface. The dissolution rate of SO_2_ is the control step of the corrosion process [20,21]. One study has demonstrated that SO_2_ combines in a lattice plane in a type of sulfur compound. The formation of Ag_2_SO_3_, Ag_2_SO_4_, and Cu_4_SO_4_(OH)_6_ is attributed to the adsorption of SO_2_ [22,23,24].

The dissolution rate is accelerated with increased HSO_3_^−^ concentration in a mixed solution containing HSO_3_^−^ and Cl^−^. HSO_3_^−^ is the dominant factor when the potential is below −0.2 V SCE, and Cl^−^ is the control factor when the potential is above −0.2 V SCE [14]. Currently, plated circuit boards (PCBs) are more widely used, but they are more sensitive to pollutants, especially SO_2_ and Cl^−^. However, almost no research has been conducted on the corrosion behavior of PCB-ImAg in a polluted environment, especially in an environment that contains Cl^−^ and SO_2_. The special environment is similar to polluted marine atmosphere environment, such as Qingdao, China. 

To study the effects of Cl^−^ and SO_2_ on PCB-ImAg, the present experiment was performed on the PCB-ImAg specimens in a salt-spray environment (the environment containing Cl^−^ only) and an acid salt-spray environment (the environment containing Cl^−^ and SO_2_) to simulate the corrosion behavior of PCB-ImAg in a polluted marine atmosphere environment. As SO_2_ dissolved into the thin liquid film to form HSO_3_^−^, some polarization experiments were conducted in a solution environment that contained Cl^−^ and HSO_3_^−^ to further explain the anti-corrosive property of PCB-ImAg in the polluted marine atmosphere environment.

## 2. Results

### 2.1. Morphology Analyses of the Specimens

**Figure 1:**
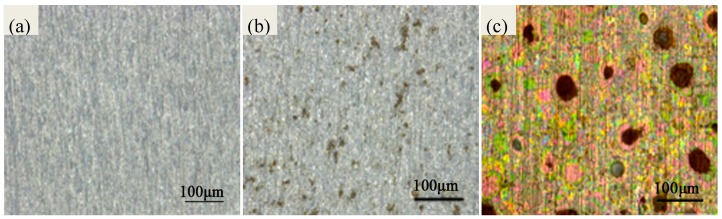
Optical micrographs of a PCB-ImAg test specimen (**a**) 0 h; (**b**) the sample in the test containing Cl^−^ only after 16 h; (**c**) the sample in the test containing Cl^−^ and SO_2_ after 16 h.

As shown in Figure 1a, the optical morphology of PCB-ImAg before the test is smooth and keeps the original color. Figure 1b shows that the color of the sample after the salt-spray test containing Cl^−^ only still keeps the original silver color. The difference is that several corrosion parts are observed on the silver surface after 16 h. The morphology of PCB-ImAg in the test containing Cl^−^ and SO_2_ is quite different from the morphology in Figure 1b. The color of PCB-ImAg has changed a lot after 16 h. The surface has been heavily damaged as shown in Figure 1c. This phenomenon shows that silver is quite sensitive to SO_2_, even with tiny amounts of SO_2_.

**Figure 2:**
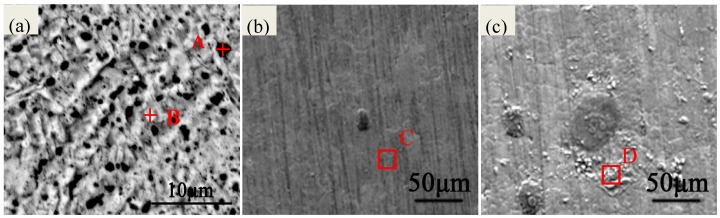
SEM morphology of PCB-ImAg: (**a**) 0 h; (**b**) PCB-ImAg in the test containing Cl^−^ only after 16 h; (**c**) PCB-ImAg in the test containing Cl^−^ and SO_2_ after16 h

Figure 2a shows the SEM (FEI Quanta 250, Hillsboro, OR, USA) morphology of PCB-ImAg before testing. Several micropores are observed on the surface where these micropores act as corrosion-active sites. Energy-dispersive spectroscopy (EDS, EDAX, Mahwah, NJ, USA) results of Figure 2a as shown in Table 1 indicate that the ratio of Ag/Cu elements is much lower in point A than the value in point B. The results show that the amount of Ag element is lower in defects; hence, the copper substrate cannot obtain good protection. Moreover, Figure 2b also shows only one bump of corrosion product in the test containing Cl^−^ only. However, the samples located in the test containing SO_2_ and Cl^−^ have suffered from more serious damage. Moreover, the EDS results of areas A and B show that the oxygen content obviously increases in the corrosion products, these results suggest more serious corrosion occurs under the environment containing SO_2_ and Cl^−^ condition.

### 2.2. Electrochemical Impedance Spectroscopy Analysis

**Figure 3:**
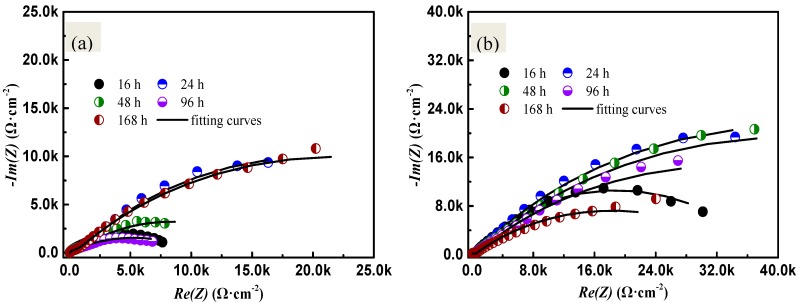
EIS and fitting curves of PCB-ImAg: (**a**) Nyquist fitting curves of the specimen after SO_2_ containing salt-spray test; (**b**) Nyquist fitting curves after the salt-spray test with no SO_2_.

**Figure 4:**
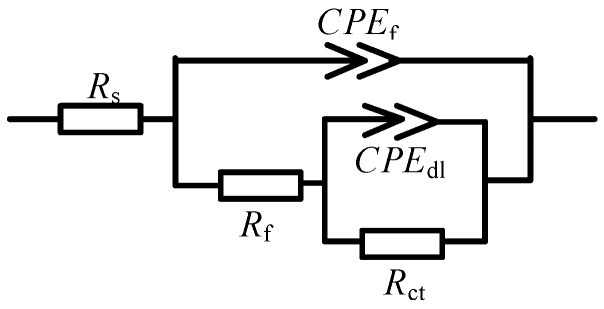
EIS equivalent circuits of PCB-ImAg specimen.

Impedance measurements were carried out for the samples after being subjected to salt tests (the test containing Cl^−^ only and the test containing Cl^−^ and SO_2_) to study the effects of SO_2_ on the corrosion behavior of sample. The EIS diagram was shown in Figure 3. The equivalent circuits presented in Figure 4 were used to fit the electrochemical impedance spectroscopy (EIS) data. R_s_ corresponds to the electrolyte resistance; CPE_f_ and R_f_ correspond to the capacitance dispersion and resistance of the corrosion products on the surface coating, respectively; and CPE_dl_ and R_ct_ represent the electric double-layer capacitor and charge transfer at the interface, respectively. The changes in the CPE_f_ and CPE_dl_ are related to the changes in the material surface. R_f_ shows the protection of the surface coating and the corrosion products. R_ct_ represents the corrosion rate; a high value corresponds to a slow rate. R_ct1_ illustrates the charge transfer of samples in the salt-spray test containing Cl^−^ and SO_2_, and R_ct2_ represents the charge transfer of samples in the salt-spray test containing Cl^−^ only. The corresponding fitting results of the EIS for PCB-ImAg under the two test environments are listed in Table 2 and Table 3.

The Nyquist plot shows two capacitive arcs: the high-frequency one conveys information on the surface coating or corrosion products, whereas the other, at low frequency, conveys information on the interface reaction. A comparison between the two figures shows that the radius in Figure 3a are always lower than that in Figure 3b, this finding suggests that SO_2_ can accelerate the corrosion of PCB-ImAg. Moreover, Table 3 indicates that R_ct1_ shows the minimal at the initial stage of the test containing SO_2_ and Cl^−^, that is, the corrosion rate is the greatest. As the extension of the salt spray test time, the corrosion rate shows a decreasing trend. However, after 24 h, the corrosion rate gradually increases. When the test time reaches 168 h, the corrosion rate decreases again. This phenomenon will be explained in the discussion part.

### 2.3. Electrochemical Measurements in Different Types of Solutions

**Figure 5:**
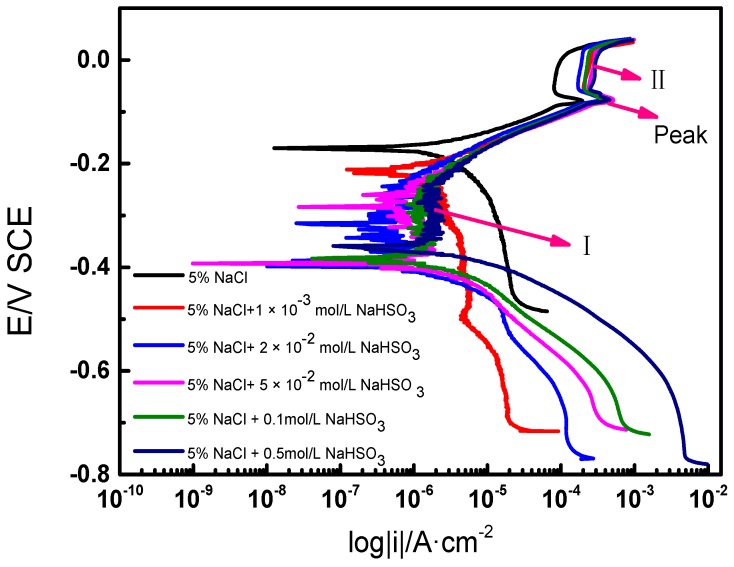
Polarization curves of PCB-ImAg in different electrolyte solutions: (**a**) 5% NaCl; (**b**) 5% NaCl + 1 × 10^−3^ mol/L NaHSO_3_; (**c**) 5% NaCl + 2 × 10^−2^ mol/L NaHSO_3_; (**d**) 5% NaCl + 5 × 10^−^^2^ mol/L NaHSO_3_; (**e**) 5% NaCl + 0.1 mol/L NaHSO_3_; (**f**) 5% NaCl + 0.5 mol/L NaHSO_3_.

The potentiodynamic polarization behavior of PCB-ImAg specimens in the 5% NaCl solution and the five other solutions containing different concentrations of NaHSO_3_ are shown in Figure 5. The cathodic current density shifts to more noble values with increased NaHSO_3_ concentration. This phenomenon indicates that cathodic reactions are accelerated by adding HSO_3_^−^ ions in the solution. The corrosion potential of the sample in the blended aqueous solution system is lower, which indicates that the samples corrode easily in the mixed solution.

All of the potentiodynamic curves have similar appearance in Region I. Corrosion has occurred in this region, and these corrosion products may have protective effects for the underlying layers. In addition, the current peak of the sample in the solution containing NaHSO_3_ is higher, which shows that the anodic dissolution is significant in this solution. The equilibrium potential of the AgCl is −0.0218 V SCE, as shown in Equation (1). This equilibrium potential deviates from the passive potential, which may be attributed to different species of corrosion ions and different concentrations of corrosion ions. Thus, Region II appearance can be attributed to the formation of AgCl [3]. The current density is high in the system that contains HSO_3_^−^ at the peak and region II labeled curve sections; these high densities show that a protective, anti-corrosion film is difficult to form on samples in mixed solutions. Thus, the polarization potential at approximately 0.02 V SCE leads to a rapid increase in current density because of the breakdown of the AgCl film. The continuous penetration of chloride ions dissolves the AgCl to form soluble AgCl_2_^−^. This reaction equation is shown in Equation (2).

(1) $$\mathrm{Ag}+{\mathrm{Cl}}^{-}\to \mathrm{AgCl}+e$$

(2) $$\mathrm{AgCl}+{\mathrm{Cl}}^{-}\to {\mathrm{AgCl}}_{2}{}^{-}$$

HSO_3_^−^ is the main controlling factor in a mixed solution when the potential is lower than −0.2 V SCE. An unstable passivation zone appears near the corrosion potential when the HSO_3_^−^ concentration is higher than 0.02 mol/L. The fluctuation of current density indicates the existence of two or more reactions. Passivation and activation alternately appear in the potential region. The current density increases when activation appears, and the current density decreases when passivation appears.

## 3. Discussions

All of the phenomena of the simulated test containing Cl^−^ only indicate that silver has strong resistance to Cl^−^; hence, the color of the silver coating shows no significant changes in a short period. The corrosion morphology shows microporous corrosion. The sedimentation of NaCl particles accelerates the formation of thin electrolyte film. Additionally, due to very thin surface coating, the surface has micro hole defects, and the defects are the corrosion-active sites [25]. Moreover, SO_2_ can induce color change on Ag [26,27]. SO_2_ dissolves in the thin electrolyte film to acidize the chemical environment, which aggravates the corrosion degree of the PCB-ImAg.

EIS measurements show that the charge transfer resistance is lower in the test containing SO_2_. Additionally, a large semicircle on the impedance spectrum is obtained in the salt-spray test containing Cl^−^ only; this result is related to the dissolution of SO_2_ in the liquid film. It is consistent with the conclusions of previous studies [21,28,29]. The charge transfer resistance of the sample under the SO_2_ and Cl^−^ condition shows initial increases, then decreases, and finally increases again. This phenomenon can be explained as follows: at the initial stage, the electrolyte containing Cl^−^ progressively permeates into the micropores under the activation effects of SO_2_ that results in the corrosion of the substrate (Cu) of nearby the micropores. Moreover, the galvanic corrosion occurs between the copper (anode) and ImAg layer (cathode). The corrosion rate was accelerated under the big cathode small anode model. Consequently, the initial corrosion rate of the sample under the salt-spray test containing SO_2_ and Cl^−^ is quite large. However, at 24 h, the corrosion products accumulated on the micropores on the sample’s surface; this accumulation hinders the penetration of electrolyte into the micropores to some extent. Accordingly, the corrosion rate decreases at that time. With the increase of test time, the corrosion process occurs on the entire surface of PCB, that is, general corrosion, which also leads to the increasing of corrosion rate. A great number of corrosion products pile on the entire surface after 168 h; hence, the corrosion rate decreases again. This result is similar to the conclusions of a previous study [30]. 

The dissolution rate of brass increases with the increase of HSO_3_^−^ concentration in a mixed solution, which contains HSO_3_^−^ and Cl^−^ [13]. In this test, the cathodic current density is accelerated by adding HSO_3_^−^ ions to the solution; these results are due to the adsorption of Cl^−^ and HSO_3_^−^ on the electrode. Lower corrosion potential and higher corrosion current density are obtained with the increase of the concentration of NaHSO_3_ due to the reaction between anions absorbed onto the electrode with the metal. The lower corrosion potential indicates that the corrosive tendency of electrodes increases. The decrease in corrosion current density is due to the formation of protective corrosion products, which hinders the invasion of ions. 

Several studies have reported the corrosion effects of Cl^−^, which showed that the diameters of chloride ions are small and have strong penetration [29,30]. The region of the current fluctuation is formed by adding HSO_3_^−^. The reaction occurs rapidly between HSO_3_^−^ and the electrode. Then, an unstable passive film forms on the surface. However, Cl^−^ ions have penetrability, and thus they react with the formed passive film. These reactions occur alternately, which explains the formation of the unstable region. An unstable passivation zone near the corrosion potential is due to the process of accelerating and inhibiting the electrode dissolution rate, which is similar to the results of another study [29]. AgCl would form under the penetration of Cl^−^, but this AgCl film would dissolve with the acceleration of Cl^−^ [3]. In this study, with the rise in potential, a more stable AgCl film was formed by the reaction of the silver coating and Cl^−^. The appearance of peak current density indicates that the AgCl film has dissolved quickly by the erosion of Cl^−^. The copper substrate loses protection from the AgCl film. Then, copper chloride forms. However, this film is less protective. Thus, the corrosion resistance of the PCB-ImAg specimen is poor in the mixed solution because an acidic environment is created by adding HSO_3_^−^, and the silver coating is sensitive to the HSO_3_^−^.

## 4. Materials and Methods

### 4.1. Design of the Experimental Setup

Salt-spray and acid salt-spray environments were simulated on a PCB-ImAg with the following structural parameters: the substrate material was FR-4 epoxy glass cloth laminate, 0.8 mm thickness, the underlying copper was 35 µm thick, and the thickness of the immersion silver which coated on Cu substrate was 0.02 µm. Before tests, the samples were dipped in acetone and cleaned with an ultrasonic washer for 10 min. Next, the samples were cleaned with deionized water for 10 min. The last step was air-drying after scrubbing with anhydrous ethyl alcohol. The samples were placed in a homemade box, and then the device was placed in two test chambers for 16, 24, 48, 96, and 168 h. The parameters of one chamber include temperature at 35 °C and salt fog concentration of 5% NaCl. SO_2_ was continuously bubbled into this chamber during the experiment. The parameters of the other chamber include temperature at 35 °C and salt fog concentration of 5% NaCl but with no SO_2_. After the salt-spray test, the samples were withdrawn and subjected to rinsing by the deionized water and dried with compressed air.

Analytical-grade NaCl, NaHSO_3_, and distilled water were used to prepare the solutions for the polarization curve test. The solution concentrations used in this experiment were as follows: 5% (wt) NaCl +0, 0.001, 0.02, 0.05, 0.1, and 0.5 mol/L Na_2_SO_4_.

### 4.2. Surface Analysis Methods

After the corrosion tests, surface analysis methods were applied, such as stereology macroscopy (VHX-2000, Keyence, Japan) and scanning electron microscope (SEM, FEI Quanta 250, Hillsboro, OR, USA).

### 4.3. Electrochemical Measurements

EIS and polarization curves were conducted using the PARSTAT 2273 electrochemical workstation (Princeton Applied Research, Oak Ridge, TN, USA). Electrochemical measurements were performed with a three-electrode cell. PCB-ImAg samples acted as the working electrodes. Platinum was used as the counter electrode, and a saturated calomel electrode was employed as the reference electrode. The available working area was 1 cm^2^. 

After the salt-spray tests, the EIS experiment on the specimens were conducted in analytically pure 0.1 mol/L Na_2_SO_4_ solution. Each set of tests was performed three times, and then Zview V3.1 (Princeton Applied Research, Oak Ridge, TN, USA) software was used to fit the EIS data.

Polarization curve analysis of the PCB-ImAg in the solution containing NaCl and NaHSO_3_ was performed. The polarization curve was measured from −0.5 V (vs. open circuit potential) with a scan rate of 0.333 mV/s. When the polarization current density reaches 1 mA·cm^−2^, the polarization curve test automatically stops.

## 5. Conclusions

The electrochemical corrosion behavior of PCB-ImAg in a polluted marine atmosphere environment (Qingdao in China) is studied through a simulated experiment. The main conclusions are as follows.

(1) Cl^−^ mainly induces the microporous corrosion. The silver coating is not sensitive to Cl^−^. Also, the settlements of salt particles on the surface of PCB-ImAg act as the corrosion-active points.
(2) SO_2_ mainly induces general corrosion of silver coating. The color of silver changes significantly under the influence of SO_2_. Then, the silver coating protection of the copper substrate decreases quickly.
(3) The lower charge transfer resistance obtained in a test containing SO_2_ demonstrates that the corrosion rate is accelerated in a mixed atmosphere.
(4) The corrosion potential is lower in mixed solutions, which indicates that the corrosion tendency increases with the increase of HSO_3_^−^ concentration. Addition of HSO_3_^−^ ions results in current fluctuation at the potential near the corrosion potential.

## Figures and Tables

**Table 1:** EDS results of A and B points in Figure 2 (at %).

Chemical Elements	Ag	Cu	O	S	C	Cl
A	26.62	73.38	-	-	-	-
B	57.91	42.09	-	-	-	-
C	38.93	45.53	0.83	-	13.56	1.15
D	17.84	33.26	42.75	0.28	5.33	0.55

**Table 2:** Impedance parameters after salt-spray tests containing Cl^−^ and SO_2_.

Experimental Period/h	*R*_s_/Ω	*Q*_dl_/Ω^−1^·cm^−2^·s^−n^	n_2_	R_dl_/Ω
16	10.30	1.831 × 10^−6^	1	9.847 × 10^3^
24	12.27	1.856 × 10^−4^	0.5660	4.823 × 10^4^
48	11.90	2.417 × 10^−4^	0.4971	1.525 × 10^4^
96	11.42	5.765 × 10^−5^	0.9799	1.048 × 10^4^
168	13.67	1.080 × 10^−4^	0.5461	4.293 × 10^4^

**Table 3:** Impedance parameters after salt-spray tests containing Cl^−^ only.

Experimental Period/h	*R*_s_/Ω	*Q*_dl_/Ω^−1^·cm^−2^·s^−n^	n_2_	R_dl_/Ω
16	9.600	7.216 × 10^−5^	0.6620	3.701 × 10^4^
24	13.24	8.508 × 10^−5^	0.5957	8.368 × 10^4^
48	9.860	6.759 × 10^−5^	0.5569	8.267 × 10^4^
96	10.01	6.956 × 10^−5^	0.5288	6.609 × 10^4^
168	11.02	7.768 × 10^−5^	0.4876	3.589 × 10^4^

## References

[1] Kim H. (2003). Corrosion process of silver in environments containing 0.1 ppm H_2_S and 1.2 ppm NO_2_. Mater. Corros..

[2] Zou S., Li X., Dong C., Ding K., Xiao K. (2013). Electrochemical migration, whisker formation, and corrosion behavior of printed circuit board under wet H_2_S environment. Electrochim. Acta.

[3] Comizzoli R., Frankenthal R., Milner P., Sinclair J. (1986). Corrosion of electronic materials and devices. Science.

[4] Wiesinger R., Martina I., Kleber C., Schreiner M. (2013). Influence of relative humidity and ozone on atmospheric silver corrosion. Corros. Sci..

[5] Tromans D., Sun R.H. (1992). Anodic behavior of copper in weakly alkaline solutions. J. Electrochem. Soc..

[6] Huang H., Pan Z., Guo X., Qiu Y. (2014). Effects of direct current electric field on corrosion behaviour of copper, Cl^−^ ion migration behaviour and dendrites growth under thin electrolyte layer. Trans. Nonferr. Met. Soc. China.

[7] Rickett B., Payer J. (1995). Composition of copper tarnish products formed in moist air with trace levels of pollutant gas: Sulfur dioxide and sulfur dioxide/nitrogen dioxide. J. Electrochem. Soc..

[8] Kear G., Barker B., Walsh F. (2004). Electrochemical corrosion of unalloyed copper in chloride media––A critical review. Corros. Sci..

[9] Warraky A.E., Shayeb H.E., Sherif E. (2004). Pitting corrosion of copper in chloride solutions. Anti-Corros. Methods Mater..

[10] Liao X., Cao F., Zheng L., Liu W., Chen A., Zhang J., Cao C. (2011). Corrosion behaviour of copper under chloride-containing thin electrolyte layer. Corros. Sci..

[11] Alfantazi A., Ahmed T., Tromans D. (2009). Corrosion behavior of copper alloys in chloride media. Mater. Des..

[12] Qiang Y., Zhang S., Xu S., Li W. (2016). Experimental and theoretical studies on the corrosion inhibition of copper by two indazole derivatives in 3.0% NaCl solution. J. Colloid Interface Sci..

[13] Liu Q., Luo H., Dong C., Xiao K., Li X. (2012). The electrochemical behaviour of brass in NaHSO_3_ solution without and with Cl. Int. J. Electrochem. Sci..

[14] Betova I., Bojinov M., Lilja C. (2013). Influence of chloride on the long-term interaction of copper with deoxygenated neutral aqueous solutions. Corros. Sci..

[15] Rahal C., Masmoudi M., Abdelhedi R., Sabot R., Jeannin M., Bouaziz M., Refait P. (2016). Olive leaf extract as natural corrosion inhibitor for pure copper in 0.5 M NaCl solution: A study by voltammetry around OCP. J. Electroanal. Chem..

[16] Sarkar N., Fuys J., Stanford J. (1979). The chloride corrosion behavior of silver-base casting alloys. J. Dent. Res..

[17] Ha H., Payer J. (2011). The effect of silver chloride formation on the kinetics of silver dissolution in chloride solution. Electrochim. Acta.

[18] Jin X., Lu J., Liu P., Tong H. (2003). The electrochemical formation and reduction of a thick AgCl deposition layer on a silver substrate. J. Electroanal. Chem..

[19] Graedel T. (1992). Corrosion mechanisms for silver exposed to the atmosphere. J. Electrochem. Soc..

[20] Changjiang Y., Chenghao L., Peng W. (2007). Investigation of the tarnish on the surface of a panda gold coin. R. Metals.

[21] Volpe L., Peterson P. (1989). The atmospheric sulfidation of silver in a tubular corrosion reactor. Corros. Sci..

[22] Odnevall I., Leygraf C. (1995). Atmospheric corrosion of copper in a rural atmosphere. J. Electrochem. Soc..

[23] Feliú S., Mariaca L., Simancas J., González J., Morcillo M. (2005). X-ray photoelectron spectroscopy study of the effect of nitrogen dioxide and sulfur dioxide on the atmospheric corrosion of copper at low relative humidity values. Corrosion.

[24] Tidblad J., Leygraf C. (1995). Atmospheric corrosion effects of SO_2_ and NO_2_ a comparison of laboratory and field-exposed copper. J. Electrochem. Soc..

[25] Pan Y., Kui X., Ding K., Gang L., Dong C., Li X. (2016). In situ investigation of atmospheric corrosion behavior of PCB-ENIG under adsorbed thin electrolyte layer. Trans. Nonferr. Met. Soc. China.

[26] Gil H., Buitrago C., Echavarría A. (2015). Characterization of atmospheric corrosion products formed on silver in tropical-mountain environments. J. Solid State Electrochem..

[27] Wan Y., Wang X., Wang X., Li Y., Sun H., Zhang K. (2015). Determination and generation of the corrosion compounds on silver exposed to the atmospheres. Int. J. Electrochem. Sci..

[28] Stratmann M., Streckel H. (1990). On the atmospheric corrosion of metals which are covered with thin electrolyte layers—I. Verification of the experimental technique. Corros. Sci..

[29] Watanabe M., Hokazono A., Handa T., Ichino T., Kuwaki N. (2006). Corrosion of copper and silver plates by volcanic gases. Corros. Sci..

[30] Olsson C.O., Landolt D. (2003). Passive films on stainless steels—Chemistry, structure and growth. Electrochim. Acta.

